# Understanding food choice: A systematic review of reviews

**DOI:** 10.1016/j.heliyon.2024.e32492

**Published:** 2024-06-05

**Authors:** Fredrik Fernqvist, Sara Spendrup, Richard Tellström

**Affiliations:** a Department of People and Society, Swedish University of Agricultural Sciences, PO Box 190, 234 22, Lomma, Sweden; b SLU Future Food, Swedish University of Agricultural Sciences, PO Box 7082, 750 07, Uppsala, Sweden

**Keywords:** Food choice, Consumer behaviour, Sustainability, Consumption

## Abstract

The topic of consumer food choice has received much attention among researchers and stakeholders within the food industry. However, in order to better facilitate food consumption towards a more sustainable direction, we need increased knowledge and understanding of factors that influence consumers’ food choice. This study is a systematic review of reviews conducted between 2017 and 2021, summarising and synthesising reviews on food choice. The aim is to provide an update of the current knowledge on consumer food choice, point out what is already known, and identify knowledge gaps to enable a prioritisation for future research. The analysis highlights decisive factors in food choice, i.e. product, available information, price, context, personal and group influences and sensory perceptions. The synthesis of findings follows a socioecological model, integrating four different levels of the social and environmental systems: the physical food environment, the social and community level, interpersonal relations and, finally, individual characteristics such as psychological and behavioural factors. Results show that behavioural patterns, influenced by strong informal institutions, such as culture and norms, can be difficult to break; for example, changing into more sustainable food behaviour. The findings suggest that more interdisciplinary research and studies in real-life settings are needed to grasp the complexity of food choice. This would allow for us to better understand consumers as social beings shaped by, among other things, the physical environment, social interactions, and culture.

## 1 Introduction

How consumers make food choices has received much attention among researchers, policy makers and stakeholders in the food industry. Recently, decision-makers have highlighted the importance of enabling and empowering consumers to make healthier and more sustainable food choices, a notion supported by other actors across the food supply chain [1]. This needed on the background that current food systems are facing numerous challenges related to e.g. public health and food security; climate change and environmental degradation; biodiversity losses; and other social, economic and environmental issues [[2], [3], [4]]. The urgent need for changing food consumption patterns have been pointed out in, for example, the “UN Sustainability Goals” [5] and in the European Union's “Farm to Fork Strategy” [1]. In response to this, and given the rich amount of research in this area, the aim of this study is to provide with an update on what is currently known on consumer food choice, and identify knowledge gaps to enable a prioritisation for future research. By conducting a review of reviews, this study enables the aggregation of a large body of research and knowledge development to be summarised and synthesised [6].

Hence, the research objective is to answer the question ‘What influences consumer food choice and how?’ The analysis applies common theoretical frameworks describing decisive factors in food choice (e.g. based on [[7], [8], [9]]), while the following synthesis of results is presented in line with a socioecological approach [[10], [11], [12], [13]]. By applying a socioecological model in the synthesis, established factors influencing food choice are reorganised to highlight different system levels in which individuals are embedded, thereby emphasising also the crucial roles played by, e.g. the food environment and culture. These theoretical frameworks are explained in the following section.

## 2 Theoretical considerations - food choice

In this study, we operationalise and define food choice as a situation where a person is shopping or purchasing food products in, for example, a supermarket, a more traditional food market, or a restaurant. This implies a choice between alternatives and follows one of the proposed concepts of food choice described by Rozin [14]. Our definition does not comprise eating at home, as the food, at that point, has already been purchased, and the meal served offers only a very limited range of choice alternatives. Additionally, ‘food intake’ is not included within the definition of food choice as it merely measures the amount of food consumed by an individual, and not how or whether this food was chosen.

Most commonly, consumer choice, including also consumers' food choice, is described as a response (dependent variable) to one or more stimuli (independent variable). This response (choice or purchase) can, in turn, be influenced by various moderating and mediating variables (e.g. Ref. [15]). Partially, this is represented by the consumer's ‘*‘black box’ whose workings can be only partially deduced*’, as described in the widely used model on consumer behaviour put forward by Kotler ([16], p. 35). This metaphor describes that each consumer makes ‘unique decisions’ based on personal experiences, knowledge, psychological characteristics, lived environment, and other factors.

It has been proposed that food choice is predominantly a learned behaviour [17], and that food preferences are influenced by learned cultural behaviours, or learning through exposure (e.g. referring to the mere exposure theory [18,19]). A significant portion of our food choices tends to be unconscious, intuitive and based on habits or heuristics [20], in contrast to choices based more on reasoning and reflection [21]. Furthermore, besides learning theories, models of food choice may be based on motivation and cognitive theories [17]. Within this latter category, the theory of planned behaviour and the theory of reasoned action (i.e. [22,23]) are mentioned as strong contributors to theory development. The complexity of food choice implies that the importance of motivational and situational (contextual) factors also needs to be considered [17].

### 2.1 Framework of analysis

There are several proposed models describing consumer food choice. Our analytical framework draws from basic elements of three commonly used food choice models: 1) Shepherd's factors influencing food preferences and choice [7]; 2) Furst et al.’s conceptual model of the food choice process [8], and 3) Brunsø’s and Grunert's depiction of consumers' food choice and quality perceptions [9] (see Table 1). Shepherd [7] presents food choice via an understanding of people's beliefs and attitudes. According to this model, psychological, economic and social factors, together with the perception of sensory attributes, lead to the formation of attitudes, which jointly, along with the purely physiological effects of food, are decisive for the final food choice. Furst et al. [8] grouped factors influencing food choice into three major components: life course, influences and personal systems. The life course includes personal roles and the social, cultural and physical environments. These factors influence ideals, personal attributes, resources, social framework and food context (e.g. setting) which, in turn, influence and shape individuals' personal systems. This includes conscious negotiations unconscious and operationalised strategies, including aspects related to e.g. sensory perceptions, monetary considerations, convenience, health and nutrition, quality and relationships. Altogether, these internal negotiations lead to food choice strategies, which are fairly stable over longer periods of time. Finally, Brunsø and Grunert [9] focus on the role of quality in preference formation, intention to buy and future purchase decisions. The concept of quality, here, is implied to be essential to food choice, as it has a double effect – sensory experiences of food are affected by personal beliefs, attitudes and values, and expected quality has an effect on food choice and purchase decisions.

**Table 1:** Basic elements influencing consumer food choice. Derived from three main theoretical conceptualisations, based on [24].

**Basic element**	**Shepherd (1989)** [7]	**Furst et al. (1996)** [8]	**Brunsø et al. (2002)** [9]
	Food, person, and economic and social factors	Life course and personal system	Before and after purchase
1) Physical product	Physical/chemical properties	Quality	Technical product specifications/sensory characteristics/Food quality
2) Available information	Availability, brand	n/a (part of value negotiation/considerations)	Intrinsic and extrinsic quality cues
3) Price	Price	Monetary considerations	Cost cues/perceived cost
4) Context	Economic/social/cultural factors	Food context	Shopping/meal preparation/eating situation
5) Personal factors	Psychological factors, mood, experiences Attitudes, beliefs	Life course/personal system Value negotiations	Implicitly assumed. Individually perceived extrinsic and intrinsic quality cues
6) Group factors	Social factors. Culture and family mentioned	Social framework, culture, family	Not explicit. Family and culture mentioned
7) Sensory perceptions	Perception of sensory attributes	Sensory perceptions	Expected and experienced quality (taste)
8) Consumer response	Food choice	Choice	Intention to buy/future purchase

From these three frameworks, the following main basic elements influencing food choice are derived, which are later used in the analysis (Table 1): First, representing different signals, or inputs, to the choice process: 1) The physical product, 2) available information about the product, 3) price and 4) context (e.g. spatial or temporal food environment). Secondly, representing individual processes in the so-called ‘consumer's black box’: 5) personal factors, 6) group factors (e.g. social interactions, family and culture), and 7) sensory perceptions. Personal factors are further divided into: a) individual behavioural and psychological determinants and b) socio-economic and demographic determinants. Finally, the response, or dependent variable: 8) food choice, implying a choice among alternatives possibly using other outcomes, such as food purchase or future purchases.

### 2.2 Framework of synthesis

**Fig. 1:**
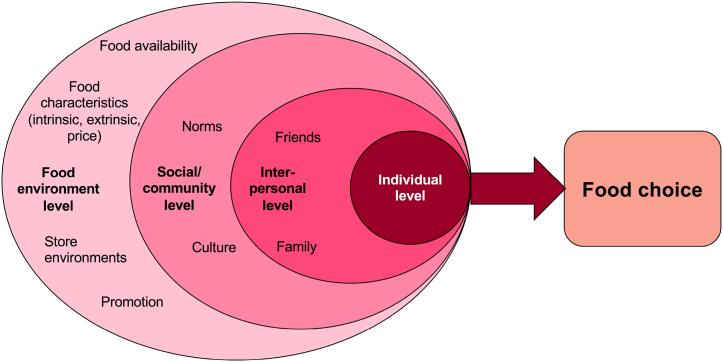
Conceptual analytical framework according to the socioecological model, based on [[11], [12], [13]].

While the analysis of the selected reviews is based on the basic elements influencing consumer food choice (Table 1), the synthesis of organising the results follows the socioecological model, originally proposed by Brofenbrenner [10,11]. This model (see Fig. 1) has previously been found to be applicable in studies of eating behaviours and food choices (e.g. Refs. [12,13]). It integrates various levels within the social and environmental systems that shape an individual's decisions. Food choice, accordingly, is influenced by the processes and characteristics at different levels, including psychological and behavioural factors. The intrapersonal level comprises the more direct relations with family, friends and other social networks. The social and community level constitutes institutions such as culture and norms, often informal depending on the context, and finally, the outer level represents the food environment. The food environment ranges from different “foodscape levels”, arguably from the national landscape (macro-level), to regional, local and domestic/meal contexts (micro-level) (e.g. Ref. [25]). Hence, the actual food product and both its intrinsic and extrinsic characteristics are, here, considered as part of the food environment at the micro-level, as highlighted by Zorbas et al. [13].

## 3 Methods

### 3.1 Study Design

The methodology of a systematic review of reviews (i.e. “umbrella review”) was followed [6]. The significant characteristic of this type of review is its focus on broad conditions that can be approached from several angles. Identified strengths include providing an easy to-use overview of current knowledge. The method enables a presentation of results that are broad, covering multiple interventions, and provides a possibility for further exploration of details [6]. The review was conducted in accordance with the Preferred Reporting Items for Systematic Reviews and Meta-analyses (PRISMA) guidelines [26].

### 3.2 Search strategy

The following five databases were searched: Scopus, ISI Web of Science (WoS) and Food Science and Technology Abstracts (FSTA), Psychinfo (Proquest) and EconLit (Proquest). The search was limited to systematic reviews published in academic peer reviewed journals between 2017 and 2021. The first search (2017–2020) was conducted on 11 October 2021 and a second search (2021) on 7 June 2022. Two members of the research team (FEF and SAS) developed and implemented the search strings in collaboration with the SLU library/information expertise. Final search strategies were adopted for the different databases and are provided in Appendix 1. Only papers in English are included, covering food of any kind. To ensure a broad and well-defined search of food, the FAO/WHO food standards [27] for food categories were applied.

### 3.3 Inclusion and exclusion criteria

Inclusion criteria were determined a priori, following the PICOS model (Population, Intervention, Comparison, Outcomes and Study Design); see Table 2. Basic inclusion criteria also stated that the paper should be: in English, peer reviewed, and published in an academic journal (not conference proceedings and book chapters).

**Table 2:** Inclusion and exclusion criteria.

Inclusion criteria	Exclusion criteria
**Population:** Healthy adult consumers (18< years) in OECD countries (or a majority of studies 50 %< made in OECD countries).	**Population:** Reviews covering the wrong population group: children and adolescents, patients and prebirth mothers. Studies with subjects in hospitals/jails and rehabilitation programmes.
**Intervention:** Nudging, information, price, general health promotion, public campaigns, buying food in store/restaurant. At least one of the variables in Table 1 (physical product, sensory properties, personal factors, group factors, context, available information or price) had to be covered by the review.	**Intervention:** Papers addressing diet, dietary intake, diet effects (CO2 emission, intake of fat, sodium, energy, fibres), medical studies and interventions, treatments, diseases linked to diets and food, e.g. addiction. Eating disorders, obesity and weight control programmes, malnutrition, diets and physical activity, dietary risks (e.g. drinking alcohol, consumption of red meat) and breast-feeding practices.
**Comparison:** Interventions influencing/studies exploring consumers' food choice. Consumers or groups of consumers should be analysed and described in terms such as gender, age and other affiliations or any variables allowing to distinguish between different consumers.	**Comparison:** Studies where there is no differentiation between consumers or groups of consumers (i.e. they are treated only as one homogenous group) are excluded.
**Outcomes:** An active food choice, purchase, selection of food, purchase, consumers making choices for themselves, or for their family/children.	**Outcomes:** There is no measurement of food choice outcomes/purchases. For example, the study focuses on other food behaviour phenomena, such as preferences, liking and experiences.
**Study design:** Systematic reviews, qualitative, quantitative and mixed methods. The research question and the inclusion criteria had to be determined before the review was carried out. The review had to contain an overview and description of included papers.	**Study design:** The study is not a systematic review. Dubious research design. No systematic presentation of results. The paper does not include a clear overview of included studies.

### 3.4 Study selection and quality assessment

**Fig. 2:**
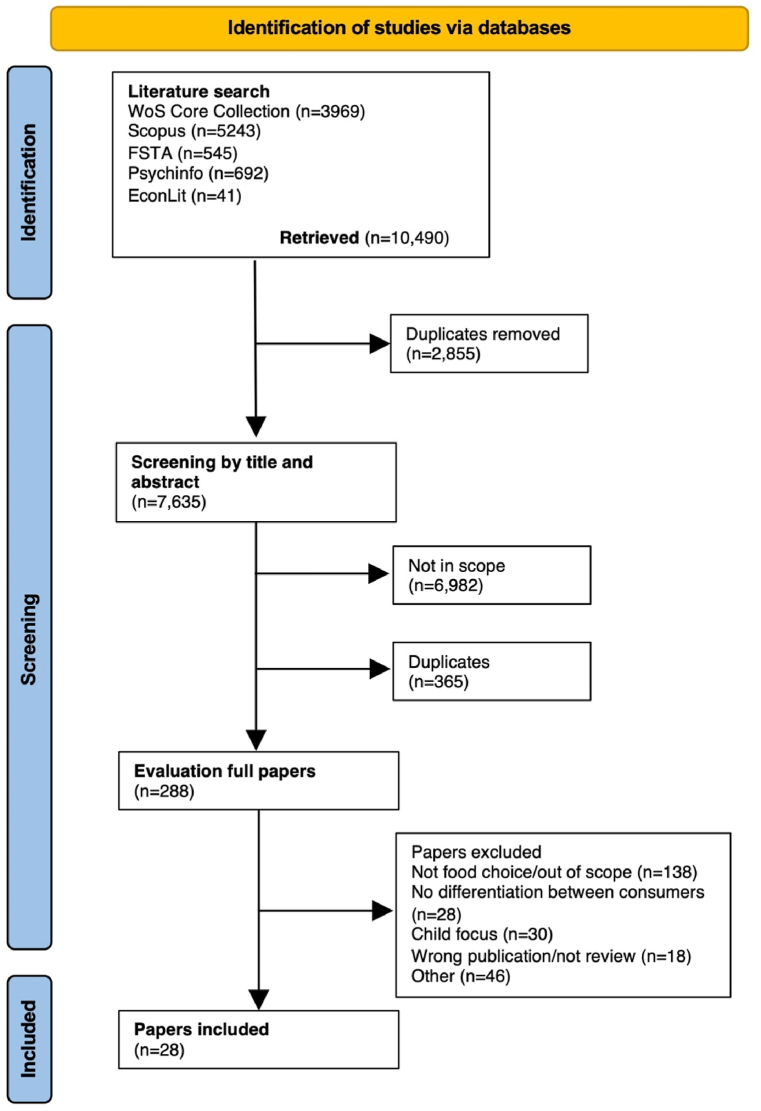
Flow diagram of the literature search.

Identified reviews were imported into Endnote, where duplicates were removed manually by FEF and SAS. Thereafter, these papers were transferred to the systematic review tool Rayyan [28], where subsequent analyses and selections were made. In order to ensure similar assessments and interpretations, and to prevent biases, the review-process was initiated by a calibration step, i.e. the researchers (FEF, SAS and RIT) conducted joint pre-readings and evaluations to ensure similar assessments of the examined papers. The initial screening of the title was conducted by SAS and FEF. Decisions regarding the abstract and, secondly, the full paper level were made in parallel by at least two researchers (FEF, SAS or RIT). Discrepancies were resolved through discussion by at least two of the participating researchers. The screening process (see Fig. 2) resulted in 28 reviews that were deemed eligible for inclusion.

The Assessment of Multiple Systematic Reviews AMSTAR-tool [29] was used in order to evaluate the scientific quality of the included reviews (included in the protocol). The AMSTAR evaluation is a measurement tool for assessing the methodological quality of systematic reviews and is based on eleven questions (each representing one point in the evaluation if fully addressed):

• Was an ‘a priori’ design provided?
• Was there duplicate study selection and data extraction?
• Was a comprehensive literature search performed?
• Was the status of publication used as an inclusion criterion?
• Was a list of studies (included and excluded) provided?
• Were the characteristics of the included studies provided?
• Was the scientific quality of the included studies assessed and documented?
• Was the scientific quality of the included studies used appropriately in formulating conclusions?
• Were the methods used to combine the findings of studies appropriate?
• Was the likelihood of publication bias assessed?
• Was the conflict of interest stated?

The included reviews showed a mean value of 7.7 (maximum value 11), with 71 per cent of the included review reporting a value more than 7. A full overview of quality ratings is provided in Appendix 2.

### 3.5 Analysis and synthesis of results

Data from the selected systematic reviews were extracted in tabular form. Firstly, study characteristics were recorded (Table 3) comprising name of authors, name of journal, time span, country where the review was conducted, included countries, study setting/context, number of articles included, method of included papers (qual/quant/mixed) and theoretical framework. Secondly, the basic elements in consumers’ food choice that were included in each review were recorded: 1) Physical product, 2) Available information, 3) Price, 4) Context, 5) Personal factors, 6) Group factors, 7) Sensory perceptions, and 8) Consumer response (see details in Table 1).

**Table 3:** Journals with included reviews.

Journal	Number of included reviews
Food Quality and Preferences	4
Trends in Food Science and Technology	4
Appetite	3
BMC Public Health	1
British Journal of Nutrition	1
Environment and behavior	1
Food policy	1
Frontiers in sustainable Food Systems	1
Global Food Security – Agriculture Policy Economics and Environment	1
International Journal of Environmental Research and Public Health	1
International Journal of Public Health	1
Meat Science	1
Nutrients	1
Nutrients	1
Nutrition Journal	1
Nutrition reviews	1
Obesity Reviews	1
Progress in cardiovascular Diseases	1
Public Health Nutrition	1
Sustainability	1

A narrative synthesis approach was deemed appropriate because of the great heterogeneity in the included systematic reviews. The implementation of analysis and synthesis of results were guided by the process presented by Popay et al. [30], in this case according to the following steps:

1) A theoretical model of how the interventions work, why and for whom. Data were extracted according to eight basic elements in food choice, Table 1.
2) Developing a preliminary synthesis. The findings were organised in line with the socioecological model [10,11], which shows the context of four different system levels leading to food choice: a) The food environment level (physical environment), b) The social and community level, c) The interpersonal level, and finally d) The individual level (Fig. 1).
3) Relationships in the data were explored, and findings and patterns of included reviews are presented, while conclusions are generalised.

## 4 Results and discussion

In total, 28 reviews were found eligible to be included. A majority were conducted in Europe (n = 19), followed by North America (n = 3) and Australia (n = 4). One review was conducted in New Zealand and one was in India. All but one covered cross-country comparisons (n = 28). All reviews were published between 2017 and 2021: 2017 (n = 4), 2018 (n = 3), 2019 (n = 6), 2020 (n = 8), 2021 (n = 7). Two reviews were based on qualitative studies, nine on quantitative, whereas a majority included studies representing a mix of methods (n = 17). Included reviews are published in 21 different journals, of which the journals Trends in Food Science and Technology (n = 4), Appetite (n = 3) and Food Quality and Preferences (n = 4) had published more than one of the included reviews (Table 3).

The included reviews cover a wide range of food categories (food in general but also special types of food, e.g. coffee, food product craftmanship, seafood); see Table 4 for an overview. Special characteristics explored included animal welfare and pasture raised livestock, meat and meat substitutes (cultured meat, plant-based food, insects), unhealthy food (e.g. snacks, cold drinks, fast food), healthy food (fruit and vegetables (F&V)), food with health properties), suboptimal food and sustainable diets (e.g. organic). The included reviews also cover a broad diversity of concepts describing food choice, at times even within the same paper. Of the included concepts, consumption or willingness/intention to consume (n = 14) as well as purchasing behaviour, purchasing decision, purchase intentions or selection (n = 13) are examples that are used. Other examples are eating (including willingness to eat and try), intake of food, and dietary behaviour (n = 9). Willingness to pay (WTP) has also been used as an indicator of choice (often in discrete choice experiments) but also ‘willingness to pay more for’ (n = 8) as well as acceptance or preferences.

**Table 4:** Main characteristics of the included reviews.

First author (year)	Number of articles included	Type of food	Terminology for food choice	Focus of the study	Method (of the studies included in the review)	Main finding (s)
Clark, B. (2017) [59]	54	Meat and fish	WTP, purchase, buy, WTB, preference, demand, choice	WTP for farm animal welfare and interventions to reduce production diseases.	Quantitative	Consumers are willing to pay a small price premium for farm animal welfare.
Hartmann, C. (2017) [55]	38	Meat and meat substitutes (cultured meat, insects)	Consumer-, consumption-behaviour	Consumer awareness of the environmental impact of meat consumption, consumer willingness to reduce meat consumption or substitute meat with alternatives, acceptance of meat substitutes and alternative proteins, e.g. insects and cultured meat.	Quantitative	Consumers are, in general, not aware of the environmental impact of meat. Large variations in consumer willingness to change meat consumption. Women more aware as well as more willing to reduce meat consumption. High meat consumption frequencies and positive attitudes towards meat associated with lower willingness to reduce consumption. Western consumers show a low level of acceptance for insects. Cultured meat less studied.
Pitt, E. (2017) [45]	30	Food in general	Food choice, consumption, intake, eat	The influence of the local food environment on food and purchasing behaviours.	Qualitative	Availability, accessibility and affordability represent key determinants of store choice and purchasing behaviours, which often resulted in less healthy food choice.
Román, S. (2017) [42]	72	Food in general	Perceptions, interest, concerns, preferences	Definitions and measurements of consumers' perceived importance of naturalness. How consumers' characteristics and attitudes towards food naturalness influence intentions and behaviour.	Mixed	Naturalness in food is important to the majority of consumers. FNI is higher among older and female consumers. Consumers with high FNI more willing to eat traditional, ecological, healthy and organic foods. Negative correlation with perception of novel food technologies.
Bryant, C. (2018) [56]	14	Cultured meat	Food choice, acceptance, buying decisions	Identify and synthesise findings of empirical studies exploring consumer acceptance of cultured meat.	Mixed	Increased familiarity, perceived feasibility, regulation, commercial availability, media coverage and ability to try are suggested as important drivers of consumer acceptance. Men and younger consumers are more positive.
Samoggia, A. (2018) [53]	54	Coffee	Consumption, purchasing, preferences, perception	Evidence and key determining factors of coffee purchasing and consumption behaviour. Synthesise the diversity of studies, methodological approaches, main issues and product types.	Mixed	Sensory qualities are key motives. Functional motives (physical and mental stimulation), health beliefs, habit, tradition and culture, connoisseurship, economic attributes (Fair trade, impact of socio-demographic variables). Context of consumption (location, occasion in time, socialising, lifestyle) is of importance.
Zorbas, C. (2018) [13]	39	Food in general, healthy and unhealthy	Healthy eating	Factors influencing healthy eating.	Qualitative	Lack of food and nutrition knowledge was a barrier to healthy eating. Food and nutrition knowledge -important to know what a healthy diet is. Supermarket availability and access to healthy foods are greater barriers to healthy eating for lower socioeconomic groups than for the general population.
Abril, E. P. (2019) [47]	14	Food in general, healthy and unhealthy	Healthy eating	Explore the effectiveness of ad campaigns in promoting consumption (go) of healthy foods or preventing (stop) unhealthy foods.	Quantitative	Findings indicate that neither stop (unhealthy) or go (healthy) information may be the most efficient tool to persuade healthy eating, but a mix of these. It was also suggested to make alternatives for more concrete (waist size) examples. Campaigns longer than six months seemed more consistently successful.
Graça, J. (2019) [43]	110	Meat and plant-based food	Food choice, behaviour, willingness	To map variables (i.e. actual or potential barriers and enablers) associated with meat reduction, meat substitution and adherence to plant-based diets.	Mixed	The male gender was associated with increased meat consumption and unwillingness to eat more plant-based diets, whereas the female gender was usually associated with lower meat consumption and with being more open to eat plant-based meals and follow plant-based diets. Urban areas, higher education, being young and higher SES more plant-based diets. The socially construed centrality of meat is a barrier.
Kushwah, S. (2019) [50]	89	Organic food	Purchase decision, intentions, consumption, WTP	Determinants of organic food consumption (purchase intentions, attitude, behaviour, WTP, preference, involvement, literacy, and decision-making heuristics).	Mixed	Identified barriers to purchase decisions are related to usage, value, image, traditional, risk and image.
Mackenbach, J. D. (2019) [60]	43	Fruits and vegetables, fast food	Dietary behaviour, intake, dietary patterns, dietary quality, food choices, food purchasing behaviour	Socioeconomic differences in the association between the food environments and dietary behaviour. Accessibility of retailers, availability or prices of foods.	Quantitative	Low SEP individuals more responsive to changes in food prices and were seen to benefit more from healthy options in a food environment. Still no clear evidence of the socioeconomic differences in the link between food environment and dietary behaviour.
Oostenbach, L. H. (2019) [41]	11	Food in general/diversity of food	Food purchase, food choice, intention	Influence of nutrition claims on knowledge, intentions, food purchases and consumption of food.	Mixed	Nutrition claims may have an impact on the knowledge of consumers with respect to perceived healthfulness, expected and experienced tastiness, and perceived appropriate portion size. Nutrition claims influence food purchase intentions, food purchases and consumption. Food with nutrition claims generally seem healthier and less tasty.
Tobi, R. C. A. (2019) [40]	30	Food in general	Preference, response, food choice	Effects on consumer food choice of three sustainable labelling schemes (nutrition, environmental, social responsibility information).	Mixed	The most preferred attribute was organic labelling. A general positive view of environmental and social responsibility food labelling schemes.
Bennett, R. (2020) [44]	16	Food in general	Consumer purchasing behaviour	The prevalence of price promotions on healthy and unhealthy foods and beverages within retail settings. The influence of price promotions.	Mixed	Price promotions more common for unhealthy foods and beverages. Findings suggest that the potential influence of price promotion is greater for unhealthy, compared to healthy food.
Cantillo, J. (2020) [38]	39	Finfish	WTP, buying, choice	Determine the most important attributes used when analysing consumers' preferences for finfish and summarise the WTP estimates of different attributes	Mixed	WTP higher for domestic products and wild, preferred over farmed foods. Higher WTP for certified products (sustainability, health and safety). Differences depending on type of label or claim.
Harbers, M. C. (2020) [63]	75	Food in general	Food choice, purchase	Evidence of the effectiveness of nudges when promoting healthy purchase and food choice in real-life food purchase environments.	Mixed	Information and position nudges may contribute to improving population dietary behaviours. Evidence investigating the moderating role of SEP was limited, although some studies reported greater effects in low SEP subgroups.
Harguess, J. M. (2020) [48]	22	Meat	Behaviour, intention, willingness	To identify factors associated with reduced meat consumption.	Mixed	Increasing knowledge alone, or combined with other methods, was shown to successfully reduce meat consumption behaviour or intentions/willingness to eat meat.
Karpyn, A. (2020) [62]	42	Healthy food	Consumer purchasing habits, choices	Explore how changes in the food retail environments (i.e. grocery and supermarket) can promote healthier food purchasing and consumption.	Mixed	Promotion was the most commonly utilised strategy for single-component interventions, and manipulating promotion, placement, and product was the most common strategy used for multi-component intervention.
Rivaroli, S. (2020) [57]	222	Diversity of food	Food choice, purchase, WTP	Key motives underlying consumer's perception of craftsmanship of foods.	Mixed	The need to assess consumers' understanding of food product craftsmanship, for policy makers and marketers in order to avoid confusion in the consumer's mind.
Stampa, E. (2020) [39]	39	Pasture-raised livestock food products	Food choice, purchase, WTP	Consumer studies on perceptions, preferences, behaviour and WTP for pasture raised products.	Mixed	Information on pasture meat production and its values can increase consumption and consumer choice.
Young, E. (2020) [58]	28	Food in general	Purchase intent, food choice, WTP, WTB	Consumer perceptions of active and intelligent packaging technologies in general and consumer perceptions of the specific technologies (including nanotechnologies).	Mixed	Familiarity is low, providing information increases trust, bridges the gap between risk and benefits and increases trust.
Bastounis, A. (2021) [49]	35	Diversity of food	WTP	WTP for food with and without eco-labels, changes due to information/attributes (certification), associations between demographics and WTP.	Quantitative	WTP higher for food with an ecolabel. Stronger effect for meat and dairy, compared to seafood, nuts, vegetables and fruits. Organic labels were valued higher compared to more specific environmental labels. Women and persons with a lower educational background had higher WTP for foods with an ecolabel. Women and younger persons more receptive to ecolabels, compared to male and older persons.
Turner, G. (2021) [61]	36	Fruits and vegetables	Consumption, intake	Assessing the dimensions of access to fruits and vegetables in the retail food environment with consumption of these products.	Quantitative	The availability of F&V is a more important component than proximity and density of food stores. The importance of physical access and acceptability was unclear. F&V affordability was not associated with intake.
Govzman, S. (2021) [46]	121	Seafood	Dietary intake, consumption	Barriers and influence on seafood consumption.	Mixed	Seafood consumers are older, more affluent, educated and physically active. Consumption relates to personal preferences, availability, cost, cooking skills, knowledge, environment, health, nutrition beliefs. Price was the dominating barrier.
Hartmann, T. (2021) [52]	40	Suboptimal food	Purchase, buy, choice	Consumers' willingness to choose/purchase/pay for suboptimal food in a retail setting, and retailers' marketing measures to support sales.	Mixed	Quality concerns have been well documented as an attitude barrier to the purchase of SF. Efficient to frame positively (sustainability, CSR, highlight “naturalness”). Contextual changes (increase availability, enhance attractiveness) support sales.
Biasini, B. (2021) [66]	67	Food in general	Behavioural intention, consumption, intake, choice	Identify main drivers of behavioural change towards sustainable diets.	Mixed	Attitude towards behaviour was identified as the most significant predictor of intention. Interventions aiming at changing intentions should target attitude and social norms, followed by perceived barriers and facilitating factors.
Potter, C. (2021) [54]	56	Diversity of food	Consumption, selection, purchase	Measure effects of eco-labels on the selection, purchase and/or consumption of any foods or drinks in both actual and hypothetical environments.	Quantitative	Ecolabels can promote the selection, purchase and consumption of more sustainable food and drinks.
Aguirre Sánchez, L. (2021) [51]	40	Sustainable food	Consumption, food purchase	Factors influencing sustainable food consumption behaviour.	Quantitative	Knowledge and attitudes showed mixed results. Sustainable consumers reported healthier lifestyles. To follow a vegetarian diet was associated with being female, non-smoker, lower proportion of daily caloric intake from fats and a lower-income.

WTB = willingness to buy, WTP = willingness to pay, FNI=Naturalness in foods, SES=Socioeconomic status, SEP = socioeconomic position, F&V = fruit and vegetables, SF=Suboptimal food.

A number of theories and concepts are applied in the reviews, e.g. food naturalness, socioecological model, capability-motivation-opportunity-behaviour, values, innovation resistance theory, choice behaviour theory, alphabet theory, value-belief-norm theory, theory of planned behaviour, willingness to pay, theory of reasoned action and social cognitive theory. However, it is worth noting that 14 reviews lack a clear connection to a theoretical framework.

The results from the analysis (the following sections) are synthesised following the conceptual framework, as depicted in Fig. 1.

### 4.1 Food environment

The food environment includes the physical environment (e.g. in store or home), availability of food (i.e. the presence of food in the particular environment) and the characteristics of the food product itself (including both intrinsic and extrinsic characteristic). A food product's intrinsic (physical food product) and extrinsic characteristics (e.g. packaging, brands, information) lead to expectations about the food, and influence food choice, as well as perceptions and liking of it [[31], [32], [33]]. In addition, credence cues or signals of trust can be explained as a specific type of extrinsic quality cue, as they cannot be easily evaluated in normal use [34]. These characteristics are often communicated in the form of labels [35] or other types of information, such as health, production methods, environmental and social orientation, local production and origin, certifications [36] and price. The physical food environment, also described as the ‘food environment’ and/or ‘nutrition environment’, may be residential areas, community, stores, or homes. At this level, food choice is often explained by concepts such as food availability (the amount, type, and quality of food a unit has at its disposal to consume) and accessibility (access to the type, quality, and quantity of food required) [37]. It is worth noting that, following these definitions, food access includes both affordability and consumer preferences. This means that a person may not access certain foods if there is no demand, or if they fall outside the ‘consumption scope’.

#### 4.1.1 Food characteristics

There is a great diversity in the applied and examined information regarding the included reviews, such as labels addressing origin, local production, organic production, fair trade, animal welfare, certification, cooking/recipes and GMO. Differences also relate to how and where the information is provided, for example, in a retail context, through promotion, advertisements or written information. Labelled products are generally preferred over unlabelled, and together with various claims, labels are mainly used to provide more information to consumers [38]. The most frequently used labels are related to health and nutritional benefits, safety, sustainability and fair-trade [38]. To be perceived as more trustworthy, information on labels must be simple and comprehensible [39]. The preferences for the product can further be influenced by its own context, as in the example of the ‘green halo effect’ – a cognitive bias effect where the product is believed to be superior due to its green characteristics [40].

##### 4.1.1.1 Health-related information

Foods carrying health and nutritional claims appear to be more likely to be chosen [39], whereas a reduced fat claim influences consumers to believe and expect the product to be less tasty [41]. Nutrition claims can, on the other hand, make the appropriate portion size appear to be larger and lead to an underestimation of the energy content of food [41]. However, nutrition and health claims do not have the same effect on all foods but differ between categories, with a higher influence on beans, eggs, fish, meat proteins, or fruit and vegetables (F&V), compared to foods high in fat and sugar [41]. Health interest was found to be positively correlated with ‘natural food’ intake [42], and health interest in general was associated with eating more meat substitutes [43].

In the retail environment, promotions for unhealthy foods and beverages are not only more frequent but also have a greater influence on purchasing decisions, compared to promotions for healthier items [44, p. 13]. Healthy products are also perceived as more difficult to identify due to a greater lack of marketing and shelf labelling, while labels are primarily used to highlight product prices and specials (of less healthy food) [45]. Moreover, the advertising of fast food in the retail environment further impacts children's requests for unhealthy foods while shopping [45].

Due to the lack of engagement among retailers in promoting healthier eating, it has been suggested that public institutions take more responsibility in promoting healthy foods [13]. It is further stressed that large-scale campaigns are important [46], and findings show that longer campaigns (over six months) have been found to be more successful in delivering health messages [47]. To add an intervention component, such as reminders via text messages, also increases the impact of information dissemination [48]. Finally, consumers tend to be more responsive to information and activities linked to assessments of their individual risks (such as measuring the waist), rather than general calls for behavioural change [47].

##### 4.1.1.2 Sustainability-related information

Research suggests that the organic label is the most well-established and valued sustainability label among consumers [49]. However, a ‘sustainability label’ itself is not necessarily sought after due to the issue of sustainability but from other associated qualities, such as availability and sensory quality. These factors are crucial motives among certain consumer groups to purchase organic food [50,51]. Buyers of ‘sustainable food’ characterise themselves as actively seeking information, for example, on how the food has been produced [52], which, in turn may significantly influence the sensory attributes of appearance and taste, leading to a higher WTP for these products [39].

Consumers associate different sustainability labels with different products; for example, coffee consumers showed a preference for fairtrade labels over ecological or organic labels [53]. Regarding environmental sustainability, research has shown that it was most effective to present GHG emissions information or claims using a combined logo and text, whereas for organic products, all formats were found to be largely effective [54]. To reduce food waste, suggested strategies to support consumers in choosing suboptimal food include using in-store messages, communicating the quality and safety of the product, offering discounts, promoting immediate consumption, using humour and humanising the product [52].

##### 4.1.1.3 Sustainability and meat reduction and the case of cultured meat

The role of information in reducing meat consumption and promoting alternatives to meat is a growing area of research. One identified barrier to reducing meat consumption is a perceived difficulty in finding practical, reliable information [43]. A study found that by adding more personal messages on a restaurant menu, compared to environmental information-only, consumption of meat was reduced [48]. Although there are several papers addressing the issue of decreasing meat consumption through various means, Hartmann and Siegrist [55] stressed that there may be an overestimation of respondents’ willingness to reduce meat consumption in general. Other effective strategies include explaining the environmental or health benefits of cultured (or “lab-grown”) meat, which increased the likelihood of individuals trying such products. In the case of cultured meat, highlighting similarities with conventional meat and presenting descriptions in a less technical manner has been found to be important in increasing acceptance of such products [56].

##### 4.1.1.4 Other information

Research findings suggest that exposure to media can influence consumption patterns in different directions, such as either supporting reduced meat consumption [43] or increasing healthy food consumption [13]. Consumers perceive a difference between hand-made and industrially produced items, often preferring handcrafted products. Industrial mass-produced food is often believed to represent a decline in quality of food, especially in terms of flavour [57]. For a specialty product like coffee, origin may be used to distinguish quality and taste [53]. Finally, packaging, as an extrinsic attribute, can significantly influence perceptions; for example, packaging that emphasises functional aspects may create the perception of a fresher taste and flavour [58].

##### 4.1.1.5 Price

The impact of price on food choice is explored in 18 of the included reviews. The major concepts applied are price (n = 10), WTP (Willingness to Pay) (n = 7), affordability (n = 4) and cost (n = 3) or a combination of these concepts (for an overview, see Table 4). Several studies highlight the connection between a low price and an increase in demand, a basic function in microeconomic theory [13,39,52,56]. The findings also show that WTP varies between different types of food. Products (with an ecolabel) such as seafood, nuts, and F&V represent a lower WTP, whereas consumers express a higher WTP for meat and dairy products [49]. Consumers also express a higher WTP concerning animal welfare [59]; however, this differs based on animal species (a lower WTP for pigs compared to cows). Food items perceived as healthy (F&V) are often perceived to be too expensive compared to unhealthy food, which are seen as both cheap and attractive [13]. Fast food was assumed to be less costly compared to home-prepared meals [13], and cultured meat was expected to be slightly higher in price compared to conventional meat [56]. However, some studies also report the opposite, where a high price is seen as a proxy for quality [57,58], e.g. in craft food products (CFP) [57] and smart packaging [58]. Moreover, culture surrounding certain products, such as a strong coffee culture, is also linked to a higher WTP [53].

Socioeconomic prerequisites matter. Higher objective food prices, higher perceived costs and lower self-reported affordability have been associated with lower diet quality or lower intake of healthy foods [60]. In contrast, when F&V are associated with increased affordability, their consumption increases [61]. For consumers with a higher socioeconomic status, cost seldom drives their purchasing decisions; instead, the choice is, to a higher extent, guided by taste and preferences for food quality [45]. Consumers who prefer ‘organic’ were also less concerned with price [40], and less price-conscious consumers were more likely to choose organic as well as other environmentally conscious options [40]. Similar patterns are identified for fish, where barriers are closely linked to cost and low income [46]. These findings imply that consumers with a limited budget tend to eat less healthy food compared to more affluent ones.

WTP was generally positive [40] for environmental attributes, such as organic labelling. Specifically, women and consumers with lower educational backgrounds tend to express a higher WTP to pay a greater price premium for foods with an ecolabel [49]. Barriers to purchasing products with sustainability labels, such as ‘fair trade’ [53] and ‘organic’ [50] are also linked to higher prices. Findings explain that while some consumers would purchase if the product was perceived as cheaper, others believe that ethical benefits justify the [higher] price [56]. Price competitiveness is thus believed to be of importance for these products to gain acceptance and market shares [56]. It has also been concluded that WTP increases when sustainability labels are combined [40,49]. Communicating local production [38] and personal benefits [39] has also been identified as increasing WTP [38]. Findings also show that WTP for animal welfare increases with income and education, is higher among younger consumers and, finally, is particularly higher among women [59]. WTP, a premium for pasture raised products and purchase intention also increased when information was provided [39]. This relationship was identified not only among consumers who were convinced by the benefits but also among those who did not strongly disapprove of the conventional practice [39]. Similar trends were identified for cultured meat, where findings show that even though the benefits/price may lead to an acceptance in principle, the product might still be rejected in practice due to a perceived high price [56].

When exploring the link between the actual type of store and perceived price, corner stores and meat markets are perceived as more expensive compared to supermarkets, chain superstores or public markets [45]. Local food stores were also perceived as taking advantage of local residents [45], and WTP decreased when buying food in supermarkets [39]. Price promotions for unhealthy foods and beverages were either more frequent or had greater influence on purchasing behaviour compared to price promotions for healthier items [44,46,62]. However, customers who received discounts purchased significantly more F&V than those who did not receive such discounts [62]. Nevertheless, as shown in Bennett et al. [44], discounts on unhealthy items increase purchases relatively more. Price promotions might also be perceived as a reward or incentive to buy [53]. Moreover, among lower socio-economic groups and students, coupons and price promotions may function as incentives to buy unhealthy foods [13].

#### 4.1.2 Local food environment

The availability of food and access to healthy and unhealthy food options in the food environment plays a key role in food purchasing decisions [45]. The local food environment mirrors the social-economic group (differences between groups) of the community, implying that consumers buy food in stores that are congruent with social status [45]. Studies explain how close and easy access to unhealthy food, such as snacks and fast food, may lead to increased consumption of such food and decreased consumption of e.g. fresh produce [45]. Unavailability [39,44,46], as well as the actual distance to access points [61] for healthy food, often results in reduced consumption. Proximity to supermarkets and outlets with less healthy products often coincides with areas of socioeconomically disadvantaged residents [13,44,60]. Here, healthy eating is limited by factors such as food insecurity, limited availability of F&V, and low access to supermarkets, but also limited transportation possibilities [13]. Walkability is a key priority for low income and minority populations [45]. At the other end, higher status supermarkets have been found to stock larger quantities of more healthy beverages compared to those targeting customers from lower socio-economic positions (groups with lower income and education) [44]. Nonetheless, Turner et al. [61] highlighted that some studies, which combined affordability, access, and sometimes availability and acceptability, have mixed findings. Accessibility is also of high importance when selecting a supermarket, with consumers favouring stores that are convenient and part of their daily routine [45]. Personal safety has also been identified as a determinant of shopping location, with people choosing to avoid stores due to reported violent incidents occurring [45].

##### 4.1.2.1 Availability and food store environment

Food availability has a key influence on purchase decisions [13]. Fruit, vegetables and meat were reported as key drivers of food store choice [45], meaning that in-store availability of these products is of high importance for why customers choose a specific store. Changes made within the food store environment can influence food choices, potentially increasing the selection of healthier alternatives or more sustainable behaviours. Practical examples include position nudges, proximity to healthy or unhealthy foods [63], special offers, appealing packaging and product layout [52]. Moving the pre-packaged produce near checkout lines has also been found to increase healthy purchases. However, displaying meal bundles has been found to be ineffective in increasing sales of healthier options [62]. Consumers representing a lower socioeconomic status tend to search for items on sale, buy in bulk, compare prices, buy store brands and cheaper cuts of meat, look for best value for money and avoid products they perceive as too expensive [45]. Factors such as limited variety and availability, poor visibility of healthy food in stores, inadequate information and low convenience are negatively associated with purchase intentions [13]. In low socioeconomic groups, less healthful in-store supermarket environments were associated with a lower dietary quality or unhealthier dietary behaviour [60]. For certain ethnic groups, it may be difficult to find traditional food items due to their limited availability. To find this type of food, the consumer is often directed to specialty shops [45]. Samoggia and Riedel [53] explain the role of atmospherics, with the example that consumers do not drink coffee in coffeehouses for the beverage itself, but for the additional lifestyle and cultural experience it offers. Finally, Zorbas et al. [13] examine the ‘weather variable’, finding that, in terms of seasonality, colder weather, compared to hotter weather, may result in a lower motivation to eat healthy for some individuals.

##### 4.1.2.2 Home environment

In the home environment, the lack of time to purchase, prepare and cook food is reported as a barrier to healthy eating [13]. Hartmann et al. [52] noted that shopping on working days might pose a barrier to purchasing some (more inconvenient, healthy) food. Limited availability of cooking facilities [13], limited usage and storage options at home have also been found to be barriers to purchasing suboptimal food [52]. Moreover, it has been explained that unhealthy food is perceived as much easier to purchase and prepare compared to healthy food [13].

### 4.2 Social level – culture and community

This section explores the impact of group factors at a social level. Following the proposal by McLeroy et al. [12], these include institutional factors, i.e. social institutions with organisational characteristics, formal (and informal) rules and regulations for the operations, and community factors, i.e. relationships among organisations, institutions and informal networks within defined boundaries.

#### 4.2.1 Culture

The term ‘culture’ is ambiguous, indicating both values and beliefs, as pointed out by Ref. [64]. According to them, one view is that culture relates to beliefs about the consequences of one's actions, and cultural beliefs are the ideas and thoughts common to several people that govern interaction. Thus, culture can be seen as an informal institution, unwritten rules of behaviour. Following the definition of culture in consumer behaviour, Solomon et al. [65], p. 580] define it as ‘the values, ethics, rituals, traditions, material objects and services produced or values by members of society’.

Culture has a major influence on food choice and may function as both a driver and a barrier when choosing food [66]. The cultural background and the culinary tradition can influence acceptance and choice of specific types of food (e.g. Refs. [46,55]), as well as cultural differences in attitudes towards body shape (body norms or beauty standards) and how this influences food choice [13]. Culture impacts what food is perceived as social and attractive. Often, alcoholic beverages and unhealthy snacks are associated with sociability, whereas F&V are not [13]. Coffee drinking is explained as a cultural experience, strongly guided by a combination of habit, tradition and culture, representing a collectively shared, symbolic object with the capacity to connect the consumer to a larger social world [53]. Cultural differences due to the country of residence were reported for aspects such as trust [58], animal welfare [59], meat consumption [55] and coffee [53]. Northern European consumers are less willing to pay a price premium for higher animal welfare products compared to those in southern Europe, which is believed to be linked to a higher trust among the northern countries in the government ensuring welfare animal standards [59].

The cultural significance of certain dishes and their links to traditions become evident when alternatives to conventional foods appear. By replacing meat with cultured meat, consumers expressed concern about losing cultural rituals, such as barbecue and Sunday roasts, and that such a change would additionally have a negative impact on traditional farmers and lead to an erosion of the countryside [56]. The concern was less common among American consumers, and the explanation was suggested to be linked to industrialisation. Americans are believed to be more used to a highly industrialised agricultural system than Europeans, and more positive to industrially produced food [56]. Craft food, on the other hand, is closely linked to a certain local cultural heritage and identity and implies a high symbolic and emotional meaning [57]. Food culture changes over time, and a ‘Westernisation’ of food culture has resulted in a transition away from healthier, traditional food practices. Zorbas et al. [13] showed that maintaining cultural cooking can have a positive impact on healthy eating, that is, if vegetables are essential components of it. Finally, culture is closely linked to messages communicated in marketing and media, which may function as a barrier to healthy eating, mainly due to the promotion of unhealthy foods [13].

#### 4.2.2 Norms

Social norms represent social standards of behaviour, indicating what people should or should not do or think under certain circumstances and may be enforced upon individuals by external pressure [67]. Institutions comprise multiple norms [68]. Hence, social norms as ‘codes of conduct’, including eating habits, have a powerful effect on both food choice and amounts consumed [69].

The special case of meat having a socially construed central position in food practice has been pointed out, and this norm may hinder a reduction in meat consumption [43]. According to Biasini et al. [66], it is important to target these social norms to support the transition towards more sustainable food consumption. A common assumption is that if consumers are more aware of environmental matters, the norms might change, thereby decreasing meat consumption. Still, this connection is not always clear, and only a few consumers seem willing to reduce meat consumption due to ecological reasons [55]. As a response, it is suggested that the change needs to be perceived as a collective reduction – a dynamic norm – highlighting how other people are important in supporting transition and learning processes [43]. If a consumer has tried and is familiar with a meat substitute, or cultured meat product, then he or she is also more positive towards these alternative products [55,56]. Cultured meat is culturally not yet acceptable, as consumers express scepticism towards cultured meat and associate it with a dystopian sci-fi like future vision, described as ‘Frankenfoods’ [56]. With proper regulation and labelling, this could partially be overcome [56]. Similarly, insect-based products are also not yet a norm, as people prefer non-insect products [55]. Choosing novel alternatives is prevented by tradition and psychological barriers due to a conflict between existing beliefs of habitual choice and the introduction of a new product [50].

The impact of norms can also be linked to social stigmatisation, influencing how cultural norms might pressure consumers to eat unhealthy foods [13]. Healthy eating habits can also contradict the male stereotype [13], suggesting gender-related norms that make it more difficult for men to choose the healthy alternative compared to women. These norms also guide what is perceived as an appropriate portion size, ultimately impacting how much food a consumer puts on the plate [41].

#### 4.2.3 Lifestyle

Lifestyles can be described as ‘a set of shared values or tastes exhibited by a group of consumers, especially as these are reflected in consumption patterns’ [65], p 585]. See also Jensen [70] for other definitions. According to Veal [71], some definitions have much in common with those of culture and sub-culture, which involve shared values and a shared way of life. Referring to Bell [72], culture is expressed through 'style of life' and is described as ‘a continual process of sustaining an identity’. Thus, lifestyle can be seen as an individual style of life that relates to culture. Lifestyle influences our eating habits, and patterns of food choice are arguably a part of a lifestyle. Examples in this review [43] describe how individuals with ‘environmental lifestyles’ were more willing to reduce their meat consumption. A ‘healthy lifestyle’ has been found to have a positive effect on purchasing healthier food [39]. Aguirre Sánchez et al. [51] also explain that sustainable consumers tend to have a healthier lifestyle, better dietary habits and enjoy food shopping. Individuals with ‘conservative’ versus ‘innovative lifestyles’ perceive novel packaging differently when making food choices [58]. A ‘busy lifestyle’ was found to be a barrier for seafood consumption, most probably due to inconvenience of preparation [58]. More detailed explanations of lifestyles were not found.

### 4.3 Interpersonal level

The interpersonal level concerns relations and interactions at the group level. It can, according to McLeroy et al. [12, p. 355], be described as ‘interpersonal processes and primary groups, formal and informal social network and social support systems, including the family, work group, and friendship networks.

#### 4.3.1 Family

The individual responsible for buying the food has a major influence on the food consumed within the family [39]. Specifically, women, to a greater extent than men, are expected to function as nutritional gatekeepers within the family [13]. Food and dietary preferences of family members also influence what food is being consumed within the family [46], such as an unwillingness to change towards a more plant-based diet through reduced meat consumption [43]. Parents’ attitudes towards food are central [46], as they are expected to function as role models, which often is perceived as difficult as it comes to responding to children's unhealthy food preferences [13].

The social networks surrounding the family can function as either facilitators or barriers to healthy eating habits through social support, food availability, preferences for healthy food, social transferability of food-related behaviours and values [13]. In families with limited resources, it is more important to ensure an adequate quantity of food for the family, rather than quality [45]. Finally, ‘food involvement’, defined as the level of importance given to food [73], has an impact on food choices. In households with low food involvement, both meat consumption and food waste are higher, and suboptimal food is consumed less frequently [52].

#### 4.3.2 Social setting

Eating and drinking are social activities closely related to the social networks surrounding a consumer [43]. To be socially included, it is important to follow acceptable ways of eating [43] as well as to buy food that is consistent with the perceived reference group [50]. Food consumption and preferences [40] are, thus, strongly guided by social value (how the perceived ability of a product provides the desired status to the buyer [50], and social desirability bias (buying food that is believed to be more socially desirable or acceptable compared to one's own thoughts or beliefs [40,74]. The social setting can also function as a ritual, such as drinking coffee with colleagues, and lead to a higher ‘social score’, facilitate friendship and interactions, and being of great importance within work environments [53].

### 4.4 Individual level

At the individual level, a great variety of personal factors are identified; see Table 5 for an overview of their distribution. The table only indicates whether each factor was mentioned within the review, but it does not specify the number of studies in which they were included, as this information was not always indicated. Factors are divided into: 1) Socio-economic and demographic determinants including gender, age, family situation, education, work or study position, income, place of residence and 2) behavioural determinants, such as attitudes, values, beliefs, risk perception, motivation, etc.

**Table 5:** Overview of personal factors influencing food choice.

Author (first author and year)	Socio-economic and demographic variables					Psychological/behavioural factors								
	Place of living	Age	Income	Education	Gender	Attitudes	Values & Beliefs	Knowledge & skills	Concerns, doubts, risk and uncertainty, behavioural control	Personal preferences	Habits	Lifestyle (and interests)	Familiarity/Experiences	Other
Clark, B. (2017) [59]	x	x	x	x	x									
Hartmann, C. (2017) [55]					x	x		x			x		x	
Pitt, E. (2017) [45]			x											
Román, S. (2017) [42]	x	x			x	x	x		x					
Bryant, C. (2018) [56]	x	x	x	x	x				x				x	VP
Samoggia, A. (2018) [53]		x	x		x	x	x			x	x		x	
Zorbas, C. (2018) [13]					x	x		x		x	x			PsE
Abril, E. P. (2019) [47]		x												
Graça, J. (2019) [43]	x	x		x	x	x	x	x	x	x	x	x		SE
Kushwah, S. (2019) [50]		x	x		x		x	x	x	x				
Mackenbach, J. D. (2019) [6]			x											
Oostenbach, L. H. (2019) [41]					x					x				
Tobi, R. C. A. (2019) [40]	x			x	x		x	x				x		
Bennett, R. (2020) [44]			x	x^O^							x			
Cantillo, J. (2020) [38]										x				
Harbers, M. C. (2020) [63]			x	x										
Harguess, J. M. (2020) [48]							x	x	x					E
Rivaroli, S. (2020) [57]		x	x	x	x	x		x	x	x	x			
Stampa, E. (2020) [39]	x	x	x	x	x	x	x	x	x		x	x	x	
Young, E. (2020) [58]	x	x	x	x	x	x			x			x	x	
Bastounis, A. (2021) [49]	x	x	x	x	x									
Turner, G. (2021) [61]	x													
Govzman, S. (2021) [46]		x	x	x	x		x	x	x			x		PsS
Hartmann, T. (2021) [52]	x	x	x	x	x	x		x	x		x		x	
Biasini, B. (2021) [66]			x		x	x	x	x	x					IdSESn
Potter, C. (2021) [54]		x	x	x	x									
Aguirre Sánchez, L. (2021) [51]			x	x	x	x	x	x	x	x				*S*
*Sum*	*10*	*14*	*17*	*13*	*19*	*11*	*10*	*12*	*12*	*8*	*8*	*5*	*6*	

V=Vegetarian, P=Political view, Ps = Other psychological factors, E = Emotions, S=Self-efficacy, O=Occupation, Id = Identity/Role identity, Sn=Subjective norm.

#### 4.4.1 Socio-economic and demographic factors

Gender stands out as one of the most used individual factors in the reviewed studies. The results for gender reveal that female consumers tend to care more about animal welfare [39,59], ‘health’ [41,66], food ‘naturalness’ [42], ecolabels [54] and ‘sustainability’ [49]. Women are also more likely to reduce meat consumption [40,43,55], follow a vegetarian diet [51], stress social motives in, for example, coffee consumption [53] and act as more active consumers [13]. On the other hand, male consumers appear to be more ‘passive’ consumers [13], maintain a higher meat consumption or willingness to eat meat [43,51,55], are more willing to consume cultured meat [56] and less prone to taking on a more plant-based diet or being vegetarian [43,51,66]. Men also seem to favour certain speciality products such as ‘speciality’ coffee [53] and consume handcrafted beers for excitement [57].

Higher income corresponds to greater consumption of fair-trade produce [53], premium products such as handcrafted food [57], seafood [46] and a higher WTP for animal welfare [39,59]. Potter et al. [54] also report that (in a majority of included studies (7 of 12), eco-label effects had higher magnitude among participants with higher incomes. Typically, lower income groups show less healthy food choices, as indicated in studies by Mackenbach et al. [60], Bennet et al. [44], Harbers et al. [63] and Aguirre Sánchez et al. [51]. Income level is also related to accessibility to healthy food, well-assorted retailers and a greater proportion of healthy choices [44,45]. Higher income consumers were also reported to contribute to more food waste [51] compared with lower income groups.

Higher education implies higher likelihood of using consumer fair-trade products [53], choosing handcrafted food [57], adopting more plant-based diets [43], higher preferences for environmental and social attributes [40], such as higher WTP for animal welfare [39,59] and openness to suboptimal food [52]. However, this is not a ubiquitous finding, as Bastounis et al. [49] showed that higher education was associated with lower WTP for sustainably labelled food, and Potter et al. [54] found various effects from eco-labelling on food choice.

#### 4.4.2 Behavioural determinants - psychological and behavioural factors

Values are goals that guide [75] and provide organisation and orientation [76] to consumers in their daily life. Closely linked to values is also the concept of beliefs, which constitute ‘*the very large number of mental or verbal statements that reflect a person's particular knowledge or assessment of something*’ [77, p. 342]. Due to the interconnectedness of these two concepts [77], the findings pertaining to values and beliefs are merged. These results show how values can significantly influence organic food purchases [50], explain meat avoidance (due to values, such as benevolence, altruism and universalism) [43], as well as higher levels of health or environmental consciousness [50] and preference towards natural food (idealism, tradition and universalism) [42]. Turning to beliefs, findings show that health beliefs influence healthy eating [13,46,53] and more sustainable eating habits (a more plant-based diet) [43].

Attitudes, defined as ‘*learned predisposition to behave in a consistently favorable or unfavorable way with respect to a given object’* [77, p. 233] are commonly assessed as predictors of consumer choice. Findings show how (positive) attitudes towards environmental concerns, animal welfare, health consciousness and food safety were associated with purchase of such products [39] as well as healthier eating [66], organic food [51], coffee [53], suboptimal food [52] and meat [43,55]. Still, it is worth noting that, despite an established use of exploring attitudes, their predictive strength in such findings is generally low due to the phenomenon known as the attitude-behaviour gap [78,79].

The link between knowledge and values/attitudes and beliefs is shown in the study by Tobi et al. [40], where findings indicate how knowledge was related to lifestyle values. Generally, studies examining consumers' knowledge and skills in relation to food choices indicate that an increased knowledge level tends to lead to ‘better’ food choices, such as reduced meat consumption [48,55], higher organic food consumption [51], and health knowledge (e.g. nutritional knowledge) as well as how it influenced healthier heating [66]. Nutrition knowledge and skills were largely believed to facilitate healthy eating, while lack of skills [43] could function as a barrier [13,46,52]. In Rivaroli et al. [57], it is further explained how seeking information and knowledge has a strong influence on consumer attitudes and beliefs. Knowledge appears to have an impact on attitude formation, which is also shown in the study by Stampa et al. [39] and Govzman et al. [46], highlighting that information and experience obtained through previous consumption generate knowledge and sensory skills about the product characteristics which, in turn, has an impact on attitude formation.

#### 4.4.3 Individual concerns and perceived risks

Several studies highlight how concerns related to risks and uncertainties function as barriers towards food and labelling. Findings related to uncertainty, health concerns, risks, distrust in the certifications/labels but also usage, value, tradition and image barriers could be of concern [50,51,66]. Additional examples [56,58] cover concerns such as ‘being unnatural’, cause environmental harm’, as well as disgust, food safety, anticipated negative sensory properties and regulation of control mechanisms. Concerns were also linked to industrially manufactured (conventional) foods, compared to less concern for handcrafted food [57]. Taken together, perceived uncertainty of negative health consequences and neophobia (aversion to novelty [58]) act as barriers for certain food choices.

#### 4.4.4 Habits and preferences

Habits represent actions that are conducted often and regularly and at times even unconsciously (Cambridge dictionary). Several studies confirm that habits indeed influence behaviour [39, 53, 55. 57, 66], and how learned habits from upbringing and childhood influence current behaviour [13]. Generally, unhealthy habits (such as smoking or drinking) imply unhealthy eating [43]. Additionally, one habit (choice of particular brands, or food waste behaviour) may have an impact on other behaviour [52].

#### 4.4.5 Individual experiences and sensory perceptions

Sensory properties, expectations and experience are addressed in 16 reviews. A common finding relates to how positive previous experiences and familiarity from consumption (i.e. learning) may have an impact on preferences [39,43,51,55,56]. On the other hand, anticipated negative sensory perceptions [46], lack of familiarity [56] and previous non-exposure can lead to barriers, such as scepticism towards a product or certain characteristics pertaining to it [52,56,58]. The link between taste and information is also explained by the fact that a reduced fat claim led consumers to believe and expect less taste compared to regular food [41]. However, through repeated exposure, it can be possible to increase positive appraisals, particularly among more neophobic consumers [43]. These findings are well in line with the ‘mere exposure’ theory, suggesting that repeated exposure to something tends to increase a preference for it [18], for example, liking of food [19].

#### 4.4.6 Psychological factors

Emotions can be described as ‘*intense feelings that often relate to a specific triggering event*’ [65], p. 581]. Zorbas et al. [13] explain how an emotional state, such as stress or boredom (together with self-perception and mental well-being), can influence healthy eating habits as well as food cravings, food addiction or eating for comfort. Similarly, negative feelings and emotional reactions, such as worry, fear and guilt towards meat and animals, have been found to be associated with reduced meat consumption [43,48].

The impact of self-efficacy and role identity (belief in one's own capacity to act) on behavioural choices (e.g. Ref. [80]) and motivations [81] is highlighted by Biasini et al. [66]. Low self-efficacy was associated with significantly higher intake of energy, fat and cholesterol. In contrast, consumers with higher levels of self-efficacy followed a healthier, more plant-based diet [51]. Additionally, psychological factors such as self-control, self-esteem, self-respect, self-regulation and motivation have been explored [13]. Finally, food consumption is part of an individual's identity, influenced by, and influencing, identity. The aspect of identity was included in Kushwah et al. [50], Rivaroli et al. [57], Biasini et al. [66] and Aguirre Sánchez et al. [51], and in all cases, it is shown to have a strong influence on food choice.

Finally, Biasini et al. [66] stated that for healthier food consumption, values and beliefs were both more relevant for food behaviour than social influences. Nonetheless, the study also stresses a lack of associations between behavioural beliefs and dietary behaviour.

### 4.5 Future research

The reviewed papers have pointed out a wide range of future research needs. Regarding the food environment, it has been found that there is a need to generate more knowledge on the influence of the food environment and aspects of health and diet-related behaviour [45,47]. It is stressed that more research is needed in realist settings to assess the complexity of food environments [48] and how multi-component interventions in this environment influences food behaviour [62]. On a more product specific level, more research is called for assessing a wider range of different types of food [38], different attributes such as the impact from information on nutritional and health claims [41,51], different labels and brands [39,52,54,57], different packaging [39,41], price promotions and price reductions [44,66] and the role of label colours and sizes [41. Also, the role of media [56], marketing strategies [44], campaigns and advertising [41,47], but also non-information nudges (e.g. availability, position, functionality and size nudges) [55,63] on food behaviour have been found to need more researched.

On the social and community level, it has been pointed out that more research is needed on how food choice is influenced in various cultural contexts [39,53] and how norms influence behaviour [13,51,52,57,58], including gender norms [51]. Future research should also further delve into examining how barriers related to sociocultural differences may be overcome to improve general health [48].

As regards the interpersonal level, very few papers took such a perspective [38]. identified a need for further investigating the influence of not being the main food purchases in the household, and [39] highlighted the need to understand the trade-off between family time and cooking time in households.

Several directions of future research covering the topic of individual level is mentioned, e.g. to explore the impact of lifestyle [51] and sociodemographic factors [39,54], such as age and gender [63]. The importance of exploring differences due to attitudes [39,51], values, beliefs [39,52] and WTP [38,57,59] are also mentioned. Other directions relate to how consumers compensate one food with another (e.g. eating a more healthy product after having eaten an unhealthy; or eating less meat after having eaten a meal with much meat) [41,55], consumer trade-offs when making choices [42] and how different types of food address concern for, e.g. novel food procucts [56]. Researchers also suggest increased study of how capability, opportunity and motivation may lead to a sustainable behaviour change [43], preferences [38], formation and expression of habits and how the influence of curiosity may have an impact on attitudes to innovation [39].

Methodological issues have also been pointed out as areas for research development, such as a need for more longitudinal studies [42,43,55,56,63] and an increase in the use of real-world settings and real market data [39,40,48,49,52,57,63]. There is also an identified request for an advancement in studies covering emerging economies and the global South [40,42,55], cross-cultural studies [56] as well as a broader diversity of countries and regions included [38,39]. Finally, the need for increased standardisations in descriptions and questions as well as coherence in conceptualisations and operationalisations [42,52,56] is identified.

## 5 Conclusion and future directions

One important contribution of this systematic umbrella review is its synthesis of our current knowledge of factors influencing food choice, drawing from the compilation of results from 28 systematic reviews. These results have been categorised according to commonly used models in consumer food choice.

Arguably, the main food choice frameworks described (i.e. [[7], [8], [9]]) consider the multifaceted influences on consumers’ food choices. However, surprisingly few of the assessed reviews addressed theoretical perspectives or frameworks of food choice. This may be a symptom of the de-contextualisation of certain aspects of food choice, and how research does not always relate to the perspective of a more complex reality. As an example, there are several reviews reporting how information on the food product may influence food choice (e.g. through a label or a piece of informative text). However, in reality, there is a great difference between actively seeking information in a purchasing situation and being provided information in an experimental setting. Therefore, the results here might be misleading, as information may not have the same impact in a real purchasing situation as suggested by the various review results.

There is a strong belief that more information will lead the consumer to make better food choices. Potentially, it could be that the consumer is overloaded by information, but as discussed by Jacoby [82], they will actually not be overloaded ‘because they [the consumers] are highly selective in how much and just which information they access and tend to stop well short of overloading themselves’ (p. 435). The consequence is that the more difficult and time-consuming it is to handle the information, the ‘less likely that the consumer will attend to some critical information’ [82, p. 435]. In reality, much of our food choices are made out of habit [20]. Consumers might rely on specific search cues when deciding on what to choose and develop strategies to simplify choices [8].

Methodologically, there has been a research drive to isolate and de-contextualise particular aspects of food choice, and test these experimentally [14]. Our review of reviews strongly corroborates this statement, even strengthening it by the fact that most studies within the included reviews were not conducted in real environments, but in surveys or experimental settings. This often implies that contextual variables, such as time and place, are not included. Indeed, food choice presumes some sort of temporal and spatial elements [14,83,84], i.e. the “when” and “where” the choice occurs – including the environment in time and space where purchasing and consumption occur. Different contexts can be the supermarket, the restaurants or other purchasing places, time of the day, weekday, weekend or holiday. Additionally, the ‘why’ question could also be more emphasised. Is the purchase intended for a specific purpose, such as a dinner, a birthday, a lunch package? Hence, in accordance with Rozin [14], there is a pressing need for research to reintegrate into the real world, i.e. into a real-life context. Although not all aspects can be assessed in any research, our knowledge development could benefit from setting the research as pieces within a larger puzzle.

A surprising finding is that only a few of the included reviews focused on sustainability issues in relation to food choice. However, in recent years, sustainability has gained increasing attention, particularly in discussions around consumers’ preferences and acceptance of meat substitutes (e.g. plant-based proteins, or insect-based food). Various actions are needed for more sustainable consumption in order to tackle current challenges, such as public health and food security, climate change and other social, economic and environmental issues (e.g. Refs. [2,3]). The necessity and urgency of these changes have been highlighted at several political and societal levels, for example as being a core concern in the UN Sustainability Goals [5] or in the EU Farm to Fork Strategy [1]. These strategies highlight the importance of enabling and empowering the consumer to make healthy and sustainable food choices, with support from stakeholders across the food chain. To foster a shift towards more sustainable food behaviours among consumers, we need to understand the complexity of food choice and understand consumers as social beings shaped by, among other things, the physical environment, social interactions and culture.

However, regarding the gender dimension, in the reviews, it is often pointed out that women often follow a more sustainable diet (both environmental and health oriented), particularly if they are well educated and live in cities. An important point of discussion here is that this recurrent finding continues to be researched abundantly. Nonetheless, how the consumption behaviour in practice may be changed in a more sustainable direction, which would be of a higher societal importance, is rarely addressed. The social value embedded in different food types and labels can prevent or support consumption of all types of food, depending on the social context surrounding a consumer. To better promote, for example, sustainable food behaviours, several interventions may be needed to influence the choices of various consumers efficiently.

It is worth putting forward some limitations of the study. Since data were extracted from the selected systematic reviews, rather than from the original primary studies, it is not possible to track specific research trends over time, analyse topic development, or identify influential researchers and research groups. Similarly, the aggregation of data in two steps when conducting a review of reviews could mean a loss in more detailed information. Also, the methodological process in conducting a systematic review imply a delay in the reporting of results. This mean that the most recent publications cannot be included here.

During the exclusion stage of screening the database searches, many reviews were excluded because they did not consider consumers as individuals or groups of individuals; instead, they treated all consumers as a single group (presenting only mean values on the whole sample and making generalisations on the whole population). Another limiting factor is how consumers are differentiated. When differences between consumers or groups of consumers are assessed, demographic variables, such as gender and age, are frequently used, reflecting their typical use as segmentation variables in consumer studies. Consumer research has long highlighted the important roles of different values and beliefs, experiences and preferences in food choices. We argue that consumers need to be understood from the perspective that they function differently. It is unlikely that everyone will respond in the same way to a certain stimulus.

Finally, do we need more of the same studies? This review of reviews shows that often basic and already well-known causalities are studied. For example, the impact of price on consumer choice, or how the socio-economic background of a consumer influences food choice, or that a healthy lifestyle has a positive effect on purchasing healthier food.

It is our conviction that for future research to enhance our understanding of food choices in real-life situations, studies must be conducted outside the experimental lab and in different contexts to address the complexity of influences stemming from temporal and situational factors. In addition, social dimensions currently appear to be less studied, particularly the role of culture and norms on our food choices. Other less studied factors influencing food choices include emotions, political views, interests and identity. Also, we identified related research areas to food choice, for example, studies on nutritional aspects and dietary intake; health issues and eating disorders; children's and youth's food behaviours. These areas were excluded from this study as they do not directly pertain to “food choice”. From a research perspective, this may indicate a need to combine different research approaches and methods from multiple disciplines to better understand consumers' behaviour and to provide more tangible recommendations to facilitate a shift towards sustainability.

## Ethics declaration

Informed consent was not required for this study, and no reason for ethical vetting was found since a review of previously published material has been made.

## Data availability statement

Data associated with included papers in the review is available in the original sources. Full supplemental data from the review process will be made available on request.

## CRediT authorship contribution statement

**Fredrik Fernqvist:** Writing – review & editing, Writing – original draft, Project administration, Methodology, Investigation, Funding acquisition, Formal analysis, Data curation, Conceptualization. **Sara Spendrup:** Writing – review & editing, Writing – original draft, Project administration, Methodology, Investigation, Funding acquisition, Formal analysis, Data curation, Conceptualization. **Richard Tellström:** Writing – review & editing, Writing – original draft, Investigation, Funding acquisition, Formal analysis.

## Declaration of competing interest

The authors declare that they have no known competing financial interests or personal relationships that could have appeared to influence the work reported in this paper.

## Figures and Tables

**Table:** 

First author (year)	Was an ‘a priori’ design provided?	Was there duplicate study selection and data extraction?	Was a comprehensive literature search performed?	Was the status of publication used as an inclusion criterion?	Was a list of studies (included and excluded) provided?	Were the characteristics of the included studies provided?	Was the scientific quality of the included studies assessed and documented?	Was the scientific quality of the included studies used appropriately in formulating conclusions?	Were the methods used to combine the findings of studies appropriate?	Was the likelihood of publication bias assessed?	Was the conflict of interest stated?	Total score
Clark, B. (2017) [59]	x	Unclear	x	x	Included yes, not excluded	x	x	x	x	x	x	9.5
Hartmann, C. (2017) [55]	x	x	x	x	Included yes, excluded not	x	Unclear	x	x	Unclear	No	7.5
Pitt, E. (2017) [45]	x	One reviewer for titles, abstracts. Full text by three authors	x	x	Included yes, not excluded	x	x	x	x	x	x	10
Román, S. (2017) [42]	x	x	x	x	Included yes, not excluded	x	x	x	x	Unclear	x	9.5
Bryant, C. (2018) [56]	x	Unclear	X	x	Included yes, not excluded	x	x	x	Unclear	x	No	7.5
Samoggia, A. (2018) [53]	x	Unclear	x	x	Included yes, not excluded	x	Unclear	x	x	Unclear	No	6.5
Zorbas, C. (2018) [13]	x	Unclear	x	x	Included yes, not excluded	x	x	x	x	Unclear	x	8.5
Abril, E. P. (2019) [47]	Unclear	Partly	x	No	Included yes, not excluded	x	No	x	x	No	x	6
Graça J. (2019) [43]	x	x	x	x	Included yes, not excluded	x	No	x	x	No	No	7.5
Kushwah, S. (2019) [50]	x	x	x	x	Included yes, not excluded	x	x	x	x	x	No	9.5
Mackenbach, J. D. (2019) [60]	x	x	x	x	Included yes, not excluded	x	x	x	x	Unclear	Unclear	8.5
Oostenbach, L. H. (2019) [41]	x	x	x	Unclear	Included yes, not excluded	Not in table	x	x	x	x	x	8.5
Tobi, R. C. A. (2019) [40]	x	x	x	x	Included yes, not excluded	x	x	x	x	Unclear	x	9.5
Bennett, R. (2020) [44]	x	x	x	x	No	No	x	Unclear	Unclear	No	x	6
Cantillo, J. (2020) [38]	x	x	x	x	No	x	No	Unclear	x	No	x	7
Harbers, M. C. (2020) [63]	x	x	x	No	No	x	x	x	x	No	x	8
Harguess, J. M. (2020) [48]	x	No	x	No	No	x	No	x	Unclear	x	No	5
Karpyn, A. (2020) [62]	x	x	x	No	No	No	No	x	x	No	x	6
Rivaroli, S. (2020) [57]	x	x	x	x	No	x	Unclear	x	x	No	No	7
Stampa, E. (2020) [39]	X	Unclear	x	No	No	x	No	Unclear	Unclear	x	No	3
Young, E. (2020) [58]	X	No	x	No	No	x	No	x	x	No	x	6
Bastounis, A. (2021) [49]	X	x	x	x	x	x	x	x	x	Unclear	x	10
Turner, G. (2021) [61]	X	Unclear	x	x	x	x	x	x	x	Unclear	x	9
Govzman, S. (2021) [46]	X	Unclear	x	x	x	x	Unclear	x	x	Unclear	x	8
Hartmann, T. (2021) [52]	X	No	x	x	Included yes, not excluded	x	No	x	x	No	x	7.5
Biasini, B. (2021) [66]	x	x	x	x	Included yes, not excluded	x	No	Unclear	x	No	No	6.5
Potter, C. (2021) [54]	x	x	x	Unclear	Included yes, not excluded	x	x	x	x	x	x	9.5
Aguirre Sánchez, L. (2021) [51]	x	x	x	Unclear	Included yes, not excluded	x	x	x	x	No	x	8.5

## Appendix 1

### Search strategy “Food choice”

#### Web of Science core collection

(TS=(Meat or dairy or vegetable* or fruit* or grain* or beverage* or wine* or beer* or cider* or bread* or fish* or seafood* or poultry or tubers or berry or berries or root* or legume* or egg* or Food* or meal* or diet* or dish* or breakfast* or lunch* or dinner* or dessert* or snack* or fat* or oil* or ice* or mushroom* or pulse* or fungi or “aloe vera” or seaweed* or nut* or seed* or confectioner* or cereal* or palm* or bakery or sweetener* or salt* or spice* or soup* or sauce* or salad* or protein* or spirit* or savoury or savory or savouries or savories))

(TS=(choice* or choose* or decision* or behavior* or behaviour* or consump* or purchase* or buy* or select*))

TS=(Consumer* Or Famil* Or Household* or individ* or person*)

TS=(systemati* NEAR/2 (review* or map* or search*))

All 4 combined, delimitation to English and 2017–2021.

#### Scopus

(TITLE-ABS-KEY (meat OR dairy OR vegetable* OR fruit* OR grain* OR beverage* OR wine* OR beer* OR cider* OR bread* OR fish* OR seafood* OR poultry OR tubers OR berry OR berries OR root* OR legume* OR egg* OR food* OR meal* OR diet* OR dish* OR breakfast* OR lunch* OR dinner* OR dessert* OR snack* OR fat* OR oil* OR ice* OR mushroom* OR pulse* OR fungi OR "aloe vera" OR seaweed* OR nut* OR seed* OR confectioner* OR cereal* OR palm* OR bakery OR sweetener* OR salt* OR spice* OR soup* OR sauce* OR salad* OR protein* OR spirit* OR savoury OR savory OR savouries OR savories)) AND (TITLE-ABS-KEY (choice* OR choose* OR decision* OR behavior* OR behaviour* OR consump* OR purchase* OR buy* OR select*)) AND (TITLE-ABS-KEY (consumer* OR famil* OR household* OR individ* OR person*)) AND (TITLE-ABS-KEY (systemati* W/2 (review* OR map* OR search*))) AND (LIMIT-TO (PUBYEAR, 2020) OR LIMIT-TO (PUBYEAR, 2019) OR LIMIT-TO (PUBYEAR, 2018) OR LIMIT-TO (PUBYEAR, 2017) OR LIMIT-TO (PUBYEAR, 2021)) AND (LIMIT-TO (LANGUAGE, "English"))

#### FSTA database

TX (Meat or dairy or vegetable* or fruit* or grain* or beverage* or wine* or beer* or cider* or bread* or fish* or seafood* or poultry or tubers or berry or berries or root* or legume* or egg* or Food* or meal* or diet* or dish* or breakfast* or lunch* or dinner* or dessert* or snack* or fat* or oil* or ice* or mushroom* or pulse* or fungi or “aloe vera” or seaweed* or nut* or seed* or confectioner* or cereal* or palm* or bakery or sweetener* or salt* or spice* or soup* or sauce* or salad* or protein* or spirit* or savoury or savory or savouries or savories) AND TX (choice* or choose* or decision* or behavior* or behaviour* or consump* or purchase* or buy* or select*) AND TX (Consumer* Or Famil* Or Household* or individ* or person*) AND TX (systemati* N2 (review* or map* or search*))

Language: English.

Years: 2017–2022.

Journal articles.

PsycInfo and EconLit (via Proquest)

(meat OR dairy OR vegetable* OR fruit* OR grain* OR beverage* OR wine* OR beer* OR cider* OR bread* OR fish* OR seafood* OR poultry OR tubers OR berry OR berries OR root* OR legume* OR egg* OR food* OR meal* OR diet* OR dish* OR breakfast* OR lunch* OR dinner* OR dessert* OR snack* OR fat* OR oil* OR ice* OR mushroom* OR pulse* OR fungi OR "aloe vera" OR seaweed* OR nut* OR seed* OR confectioner* OR cereal* OR palm* OR bakery OR sweetener* OR salt* OR spice* OR soup* OR sauce* OR salad* OR protein* OR spirit* OR savoury OR savory OR savouries OR savories) AND (choice* OR choose* OR decision* OR behavior* OR behaviour* OR consump* OR purchase* OR buy* OR select*) AND (consumer* OR famil* OR household* OR individ* OR person*) AND (systemati* and (review* OR map* OR search*))

Delimitation to 2017–2021.

## Appendix 2

AMSTAR evaluation of included reviews (X = yes).
